# Regulation, targets and functions of CHK

**DOI:** 10.3389/fcell.2022.1068952

**Published:** 2022-12-09

**Authors:** Shudong Zhu, Rong Sun, Xialing Guo, Yuanwu Bao, Dianzheng Zhang

**Affiliations:** ^1^ School of Medicine, Nantong University, Nantong, China; ^2^ Argus Pharmaceuticals, Changsha, China; ^3^ Triapex Biotechnology, Shanghai, China; ^4^ Department of Bio-medical Sciences, Philadelphia College of Osteopathic Medicine, Philadelphia, PA, United States

**Keywords:** CHK, CSK, Src, protein tyrosine kinase, oncogene, cancer

## Abstract

Src family kinases (SFKs) play pivotal roles in multiple signaling pathways (Yeatman, 2004). SFK activity is inhibited by phosphorylation at its C-terminal tyrosine, by CSK (C-terminal Src kinase) and CHK (CSK-homologous kinase). CHK expression is restricted to normal hematopoietic cells, brain, and colon tissues. Downregulation of CHK in brain and colon tumors contributes to tumorigenicity in these tissues. CHK does not phosphorylate Src efficiently, however, in contrast to CSK, CHK inhibits Src kinase activity allosterically. Although the functions of CHK are still largely unknown, potential substrates of CHK including β-synuclein, α-tubulin, α-spectrin, 14-3-3, and Hsp90 have been identified. CHK is regulated epigenetically *via* promoter methylation. As the unknown roles of CHK are beginning to be revealed, current knowledge of regulation, molecular targets and functions of CHK is summarized, and important topics for future CHK research are discussed.

## 1 Introduction

Src is a kinase that plays pivotal roles in many signaling processes (Yeatman, 2004). CSK (C-terminal Src kinase) phosphorylates Src at its C-terminal tyrosine (Y530 in human) (Avraham et al., 1995; Chong et al., 2004), inactivating Src, playing a key role in controlling tumorigenic properties of Src and other physiological processes.

Human CHK (CSK-homologous kinase) is a kinase composed of 527 amino acids that has highest homology with human CSK, sharing 53% amino acid identity. The genomic structure of CHK is identical to that of CSK as reflected in the organization of its exons (Hamaguchi, et al., 1994). CHK has also been reported by different research groups as HYL, MATK, CTK, LSK, NTK, and BATK (Bougeret et al., 2001). Similar to CSK, CHK consists of SH2, SH3 and kinase (SH1) catalytic domains.

However, there is evidence to indicate that CHK and CSK have different biological roles. For example, CHK expression in brain increases postnatally whereas the CSK expression decreases with age (Hamaguchi et al., 1996). While similar to CSK, CHK has been shown to phosphorylate and inactivate Src, it can also inhibit Src in a phosphorylation-independent mechanism, different from CSK (Chong et al., 2004).

Moreover, potential substrates such as β-synuclein have been identified for CHK, specifically (Ia, et al., 2011). Expression of CHK has been shown to be regulated epigenetically recently (Chüeh et al., 2021; Zhu et al., 2021). However, the physiological and pathological roles of CHK as well as the regulation of CHK are still largely unknown.

In this review, we summarize the current knowledge about CHK, including regulation, molecular targets of CHK, as well as its biological functions, especially in the development of cancer. We also discuss challenging tasks in this field and its promising future.

## 2 Structure and isoforms of CHK

**FIGURE 1:**
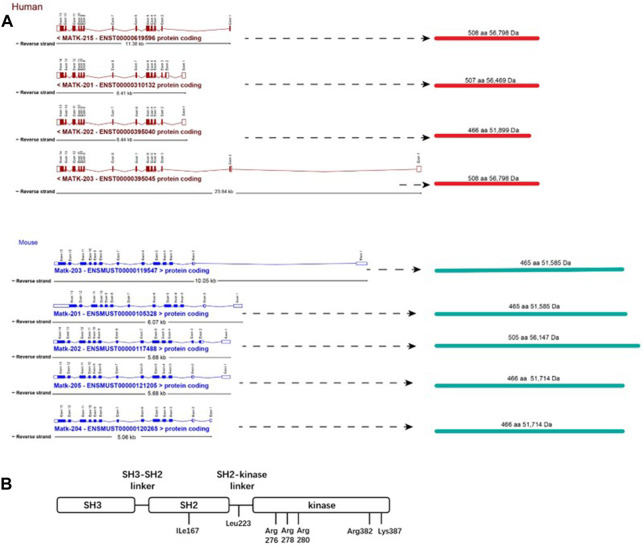
Structures of CHK.**(A)** Gene structure and isoforms of CHK. Boxes correspond with exons. Unfilled boxes at the ends of the transcripts represent untranslated regions (UTRs). Lines represent introns. aa, amino acid; Da, Dalton. Merging gene and transcript models of CHK were from ensembl database (https://uswest.ensembl.org/index.html) (Human:GRCh38.p13; Mouse:GRCm39).**(B)** Structure of CHK protein. CHK consists of SH2, SH3 and kinase domains, and lacks a myristoylation signal and all the regulatory amino acids present in SFKs, including the autophosphorylation (Y419), and the carboxyl-terminal tyrosine (Y530). Ile167 is important for efficient and specific binding of CHK to its substrates, determining the functional differences among the three SH2 domains of CHK, CSK (Glu127) and Src (Lys 200) (Ayrapetov et al., 2005). Leu223 in the SH2-kinase linker region is important for maintaining the full catalytic activity of CHK (Mikkola Bergman, 2003). The kinase domain contains amino acids determining the tight binding of CHK/SFKs and inhibition of SFKs by the non-catalytic mechanism. The basic residues including Arg276, Arg278, and Arg280 (in αD helix) and Arg382 and Lys387 (in αF-αG loop) are important for the catalytic activity of CHK. Among these residues, Arg382 and Lys387 have mild effects on the affinity of CHK for SFKs (Advani et al., 2017).

The human and murine CHK gene codes for four and five splicing mRNA isoforms, respectively. However, CHK proteins of only two molecular weights (52 kDa and 56 kDa) are produced in each species (Zhu et al., 2008) (Figure 1A). The 52 kDa CHK isoforms are predominantly expressed in mouse, whereas the 56 kDa isoforms are predominantly expressed in human (Chow et al., 1994a; Zhu et al., 2008). Besides the similar structure in SH1, SH2 and SH3 domains, both CSK and CHK lack a myristoylation signal, the autophosphorylation site (Y419), and the carboxyl-terminal tyrosine (Y530), all present in Src (family members) (Figure 1B).

On the other hand, although CHK protein is homologous to CSK, CHK has a unique N-terminus, and its SH3 and SH2 domains share only 30% and 59% amino acid identity approximately to that of CSK, respectively (Hirao et al., 1997). In addition, part of the structural basis for the functional difference between SH2 Domains of CHK and CSK has been determined, including Glu127 in CSK, and Ile167 in CHK (Ayrapetov et al., 2005). Therefore, CHK has unique structure that supports its potentially different biological roles from CSK.

## 3 Expression of CHK

For many years, expression of CHK is believed to be limited in the brain, and in most types of hematopoietic cells of bone marrow, spleen and thymus (except erythroid cells) (Hamaguchi et al., 1996), and weakly expressed in the testis (germ cells) (Chow et al., 1994b; Kaneko et al., 1995). Surprisingly, many years after, we found that CHK was also expressed in normal colon tissues (Zhu et al., 2008), and Clemmons lab found that CHK was also expressed in smooth muscle cells (Radhakrishnan et al., 2011).

The restricted expression of CHK to certain tissues, in contrast to the ubiquitous expression of CSK (Okada et al., 1991), suggests specific roles of CHK in certain tissues. Besides, expression of CHK and CSK may differ developmentally, or with the same cytokine stimulation: in the developing mouse brain, expression of CHK increases postnatally, while the expression of CSK decreases with age (Brinkley et al., 1995); in human monocytes, IL-4 and IL-13, but not IFN-γ, induce CHK, in contrast to CSK (Musso et al., 1994). The different expression of CHK in comparison to CSK also suggests different roles CHK may play.

## 4 Functional studies from CHK knock-out animals

CHK knock-out mice appeared to be normal (Hamaguchi et al., 1996), in contrast to the CSK knock-out mice, which is embryonic lethal due to defects in neural tube formation (Imamoto and Soriano, 1993; Nada et al., 1993). CHK deficient mice also showed normal hematopoiesis. This includes blood counts, white cell differential counts, bleeding tendency, the sizes of spleen and thymus, the distribution patterns of hematopoietic stem cells, monocytes/macrophages, B-cells, and T-cells, the colony formation of bone marrow and spleen cells, as well as megakaryocyte counts in bone marrow (Hamaguchi et al., 1996; Samokhvalov et al., 1997). The absence of severe abnormalities in phenotypes in CHK knock-out mice is likely due to the compensatory effects of CSK, which is ubiquitously expressed. However, a more extensive study found that stimulation of the hematopoietic cells of these mice with IL-7, or injection of these mice with antigen (TNP-ovalbumin), led to physiologic responses, very different from that of CHK+/+ mice, suggesting that CHK may be involved in certain immune responses that CSK is not capable of (Lee et al., 2006).

## 5 Expression of CHK in cancers

### 5.1 Breast and pancreatic cancers

Immunochemistry showed CHK expression in most primary invasive breast ductal carcinomas, but not the adjacent normal tissues from the same patients (Zrihan-Licht et al., 1997).

On the other hand, it has also been shown that CHK suppressed HRG-mediated signaling pathway and oncogenic properties of breast cancer cells (Bougeret et al., 2001). Although the roles of upregulation of CHK in the development of breast tumors is unknown, the mechanism that trastuzumab induces CHK mediated ErbB2 degradation, leading to the inhibition of breast cancer cell growth may be used as a novel strategy to treat ErbB2-positive breast cancers (Dokmanovic et al., 2014).

Similarly, CHK appears to be expressed in pancreatic cancer but not in normal tissues (Fu et al., 2006). CHK also binds to ErbB-2 in PANC-1 cells and suppressed EGF-stimulated Lyn activation, inhibiting cell invasion. This also supports therapeutic approaches based on CHK to inhibit EGF triggered signaling for the treatment of pancreatic cancer.

### 5.2 Brain and colon cancers

CHK is expressed in human neurons, astrocytes and oligodendrocytes, but not in neuroblastoma, astrocytoma or glioblastoma tumors. Consistently, CHK overexpression in these CHK deficient cells suppresses growth and proliferation of these cells. These findings suggest that loss of CHK expression may play a role in the tumorigenesis of brain cells (Kim et al., 2004).

**FIGURE 2:**
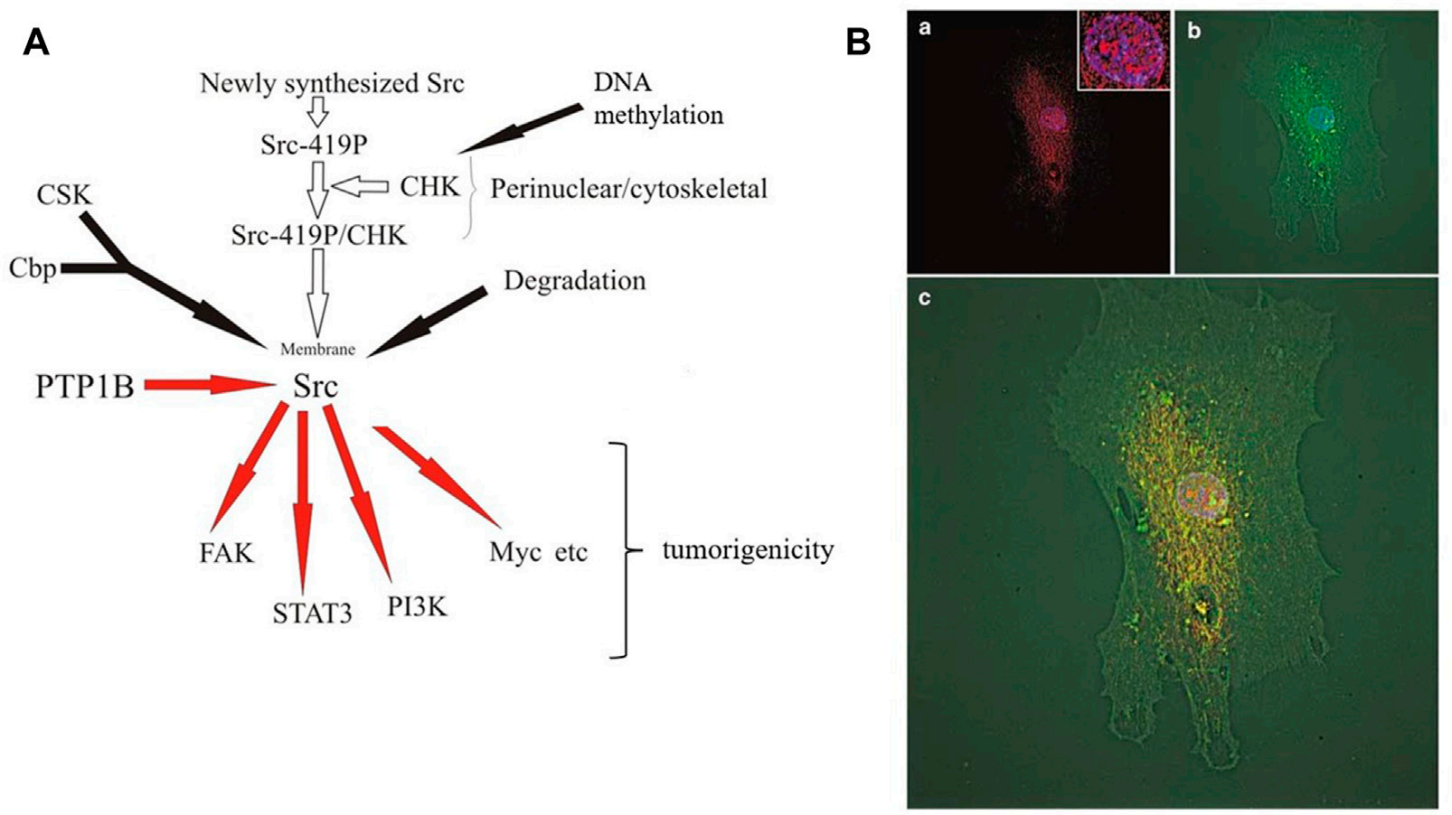
CHK in colon cells.**(A)** Regulation and function of CHK in colon cancer cells. Newly synthesized Src is autophosphorylated first, followed by binding to CHK at perinuclear region and Src becomes inactive (Zhu et al., 2008). Inactive perinuclear Src transits to the plasma membrane through the cytoskeleton and loses its interaction with CHK at membrane and becomes active. Active Src induces phosphorylation of Cbp in the membrane, which recruits CSK to the membrane in the proximity of Src (Kawabuchi et al., 2000). Src becomes phosphorylated by CSK at Y530 and stays inactive. Under appropriate conditions, PTP1B etc. starts to dephosphorylate Src at Y530 and to activate Src (Zhu et al., 2008). Active Src then phosphorylates its substrates and initiates positive signaling pathways. Activated Src also triggers activation-dependent ubiquitination and subsequent degradation of Src as negative feedback (Oda et al., 1999; Pan et al., 2011). In colon cancer cells, the loss of CHK expression allows Src to keep active at perinuclear region, leading to the increase of tumorigenicity (Zhu et al., 2008).**(B)** Subcellular localization of CHK and Src in FHC cells. CHK is localized in the nucleus, perinuclear region and plasma membrane. CHK is colocalized with Src at perinuclear region. Normal colon epithelial FHC (Fetal Human Colon) cells were immunostained for CHK and Src and microscopic fluorescence images were deconvoluted. **(a)** Superimposing of CHK (red) and 4,6-diamidino-2- phenylindole (DAPI) (blue) staining. Inset, same staining of the nucleus with high magnification. **(b)** Superimposing of Src (green) and DAPI (blue) staining. **(c)** Superimposing of CHK (red), Src (green) and DAPI (blue) staining (Zhu et al., 2008).

While CHK is expressed in normal colon cell lines, CHK protein levels are significantly decreased in various colon cancer cell lines. Likewise, while CHK is also expressed in normal colon tissues, its expression is greatly decreased in colon cancer tissues from the same patients (Zhu et al., 2008). The decrease of CHK expression results in Src activation without affecting the level of Src phosphorylation at Y530, and enhanced the tumorigenicity including anchorage-independent cell growth and cell invasion of colon cancer cells (Zhu et al., 2008; Figure 2A). The seemingly paradoxical upregulation of CHK in some cancer types but downregulation in other cancer types suggests that CHK may act as a tumor suppressor or proto-oncogene depending on the cellular context.

## 6 Subcellular localization and cellular roles of CHK

CHK is often recruited to the plasma membrane *via* binding to ErbB2, c-Kit and TrkA.

In breast cancer cells, CHK has been shown to be associated with ErbB-2 (*via* CHK SH2 domain) upon heregulin stimulation (Zrihan-Licht et al., 1997). This leads to the suppression of Src kinase activity by CHK, followed by the attenuation of the activated receptor signaling and oncogenic properties (Zrihan-Licht et al., 1997; Bougeret et al., 2001; Kim et al., 2002).

In megakaryocytic cells, CHK is translocated to the plasma membrane by association of its SH2 domain to autophosphorylated Tyr^568/570^ in c-Kit upon stimulation by stem cell factor/kit ligand (SCF/KL) (Price et al., 1997). By associating with c-Kit, CHK is likely to suppress activity of SFK (which also binds activated c-Kit), and the signaling pathways involving PI3K, PLCγ-1, and ras-GAP (Jhun et al., 1995). c-Kit signaling is important for hematopoiesis, and some other functions (Lennartsson and Rönnstrand, 2012). In the megakaryocytic cell line Dami, elevated CHK expression is able to suppress activation of Lyn kinase and VLA5-mediated Dami cell spreading that is dependent of Lyn activation (Hirao et al., 1998).

In neuronal cells, CHK was found to be involved in TrkA signaling. CHK, *via* its SH2 domain, is associated with phosphorylated TrkA receptors (Tyr-785) upon NGF stimulation. CHK overexpression activates the mitogen-activated protein kinase (MAPK) pathway upon NGF stimulation, and contributes to the neurite outgrowth of the cells (Yamashita et al., 1999). Further studies have shown that CHK induces formation of a complex containing SHP-2 and Grb2, leading to the activation of Ras and Raf/MEK/MAPK pathway. Interestingly, activation MAPK by CHK was independent of presence of NGF or the inhibition of Src kinase by CHK (Zagozdzon et al., 2006).

In colon epithelial cells, CHK colocalizes with Src at perinuclear region (Zhu et al., 2008). There is evidence to suggest that newly synthesized Src is autophosphorylated first, followed by binding to CHK and thus Src becomes inactive at perinuclear region, where it is associated with cytoskeleton structure (Zhu et al., 2008; Figure 2). Inactive perinuclear Src transits to the plasma membrane through the cytoskeleton structure and loses its interaction with CHK and becomes active. Active Src induces phosphorylation of Cbp in the membrane, and phosphorylated Cbp recruits CSK to the membrane in the proximity of Src (Kawabuchi et al., 2000). Src becomes phosphorylated by CSK at Y530 and stays inactive. Under appropriate conditions, PTP1B etc., starts to dephosphorylate Src at Y530 and to activate Src (Zhu et al., 2008). Active Src then phosphorylates its substrates and initiates positive signaling pathways. Activated Src also triggers activation-dependent ubiquitination and subsequent degradation of Src as negative feedback (Oda et al., 1999; Pan et al., 2011). In colon cancer cells, the loss of CHK expression allows Src to be active at perinuclear region, leading to the tumorigenicity (Zhu et al., 2008).

Besides its perinuclear localization, we have also shown nucleus localization of CHK in colon cells (Zhu et al., 2008; Figure 2B). This has confirmed earlier report that overexpression of CHK in COS-1 cells showed nuclear localization and growth inhibition of CHK (Yamaguchi et al., 2001). In human immature myeloid KMT-2 cells, Lyn and overexpressed CHK are associated with mitotic chromosome scaffolds and spindles. CHK overexpression induced a decrease in Lyn activation and phosphorylation of some associated proteins. CHK appears to participate in metaphase chromosome dynamics, since CHK overexpression caused aberrant chromosome movement. Probably due to insufficient formation of mitotic spindles, this leads to multinucleation, causing perturbation of normal cell division process, eventually leading to growth inhibition (Yamaguchi et al., 2001). Expression of CHK in the nucleus prolonged S phase of the cell cycle along with the nuclear multi-lobulation, suggesting that S phase may also be involved in the growth inhibition (Nakayama and Yamaguchi, 2005). Further experiments have shown tyrosine phosphorylation of a variety of proteins in the nucleus at the tyrosine residues upon CHK expression, suggesting there are probably potential substrates of CHK in the nucleus yet to be identified, besides SFKs (Nakayama et al., 2006). Structurally, N-terminal unique domain of CHK appears to be involved in the enhanced protein tyrosine phosphorylation in the nucleus and the induction of the multi-lobulation (Nakayama and Yamaguchi, 2005; Nakayama et al., 2006).

In addition, CHK could also relocate to cytoskeleton from the plasma membrane, which happens in platelets upon thrombin stimulation (Hirao et al., 1997), suggesting a role of CHK in platelet activation.

Last but not least, the level of CHK is regulated during development of germ cell, suggesting that CHK may be involved in the regulation of differentiation of male germ cells (Kaneko et al., 1995). In summary, CHK can be recruited to the nucleus, perinuclear region, and plasma membrane to perform different roles under different circumstances.

## 7 Molecular targets of CHK

### 7.1 Src family kinases

#### 7.1.1 *Via* phosphorylation

Like its homologue CSK, CHK has been shown to phosphorylate the C-terminal regulatory tyrosine of Lck (Chow et al., 1994b) and Lyn (Hirao et al., 1997, 1998; Chong et al., 2004; Klages et al., 1994), both are hematopoietic members of the SFK. However, Yes kinase is not phosphorylated or inhibited in the presence of CHK overexpression, demonstrating the difference in the specificity of CHK for different Src family members (Yamaguchi et al., 2001). CHK also inactivates Src kinase (Davidson et al., 1997; Chong et al., 2004) and Fyn kinase through phosphorylation (Davidson et al., 1997) *in vitro*. When expressed in CSK deficient fibroblasts of mouse embryo, CHK downregulates Src kinase activity (Davidson et al., 1997). Moreover, CHK is able to phosphorylate Src at its C-terminal *in vitro*, accompanied by Src inactivation (Advani et al., 2017). These lines of evidence indicate Src is a physiological phosphorylation substrate of CHK kinase.

In CSK-deficient fibroblasts, CHK reduced the activity of Src family kinases, but CHK was not capable of suppressing antigen receptor-signaling in a T-cell line, suggesting that CHK functions differently in different cellular contexts (Davidson et al., 1997). On the other hand, the negative regulation of Src family kinases by CHK has been shown with a preferred selectivity toward Lyn but not Src in platelets (Hirao et al., 1997). This probably is due to the association of Lyn, but not Src, with CD36, suggesting differential targets CHK may be aimed in different cellular contexts.

#### 7.1.2 Allosteric

Besides inactivation of Src *via* phosphorylating its C-terminus, CHK can also inhibit the activity of SFKs by a non-catalytic mechanism (Chong et al., 2004, 2006). This has been confirmed by results of immunofluorescence microscopy showing that CHK colocalizes with Src in normal colon epithelial cells in addition to the co-immunoprecipitation, Src inactivation and phosphorylation results (Zhu et al., 2008) (Figure 2B).

Further biochemical studies have indicated that CHK is relatively weaker in phosphorylating SFK C-terminal but is a strong, non-catalytic, allosteric inhibitor, binding SFKs with high affinity, leading to efficient inactivation of SFKs, in comparison to CSK. Some of the major motifs or residues controlling CHK’s high affinity binding and inhibition of SFKs have also been determined (Advani et al., 2017; Figure 1B).

### 7.2 Src homology 2 domain containing protein tyrosine phosphatase substrate-1 (SHPS-1)

SHPS-1 is a transmembrane scaffold protein often to recruit adaptive surviving signaling proteins upon IGF-I stimulation in response to hyperglycemic stress in vascular smooth muscle cells (VSMCs). Forming SHPS-1 complex facilitates activation of phosphatidylinositol 3-kinase (PI3) and MAPK pathways and subsequent cell proliferation and migration.

In VSMCs, after IGF-I stimulation, IGF-IR phosphorylates SHPS-1 at Y469/495, allowing CHK binding to IGF-IR, and CHK subsequently phosphorylates SHPS-1 at Y428/452, which is important for IGF-I stimulated cellular proliferation (Radhakrishnan et al., 2011).

### 7.3 Paxillin

Paxillin is a cytoskeletal protein playing important roles in focal adhesions. Paxillin also binds to Src *via* Src SH3 domain and is a Src kinase substrate. Using T cell as a model system, paxillin was identified in the CHK immunoprecipitants (Grgurevich et al., 1999). Consistently, CHK and paxillin also colocalize in subcellular fractions. Further experiments show that CHK binds directly to phosphorylated but not the unphosphorylated paxillin, *via* SH2 domain of CHK (phospho-tyrosine binds arginine in the FLVRES motif) (Grgurevich et al., 1999). Since CHK reduced phosphotyrosine levels of paxillin, it is likely CHK affected phosphorylation of paxillin indirectly, possibly through downregulating activity of kinase(s) such as SFK. Combined evidence suggests that it is possible that besides the role of paxillin at focal adhesions in cytoskeleton remodeling, part of the cytoskeletal paxillin is first phosphorylated at perinuclear region by active Src newly synthesized, followed by CHK binding to the phosphorylated paxillin, therefore paxillin probably brings CHK to the close proximity of Src to keep Src inactive before being recruited to the plasma membrane (Zhu et al., 2008; Figure 2; Grgurevich et al., 1999; and Turner et al., 1990).

### 7.4 Synuclein and other potential CHK targets

Ia et al. (2011) used the kinase substrate tracking and elucidation (KESTREL) technique to search for potential physiological CHK substrates from cytosol extract of rat brain. The experiment has identified β-synuclein, α-tubulin, α-spectrin, 14-3-3, and Hsp90 as potential substrates of CHK. Among them, the rate of phosphorylation suggests that β-synuclein is a preferential substrate of CHK kinase.

The *in vitro* kinase assay in presence of CHK and recombinant β-synuclein or its mutants also identified tyrosine 127 in β-synuclein as the preferential phosphorylation site. The fact that CHK Phosphorylates β-Synuclein and SFK with almost same efficiency *in vitro* and that CHK phosphorylates β-Synuclein in CHK transfected cells further support β-Synuclein as a physiological substrate of CHK. However, the functional significance of this phosphorylation by CHK has not been identified yet.

Using a peptide library, the authors have also defined the optimal phosphorylation motif of the substrates recognized by CHK as E-x-[Φ/E/D]-Y-Φ-x-Φ (Φ: hydrophobic residue; x: any residue) (Ia et al., 2011). It is very likely that further research will establish novel physiological targets of CHK besides SFKs.

## 8 Regulation of CHK expression

Whereas CSK is constitutively expressed, CHK expression is induced in T cells upon activation (McVicar et al., 1994). CHK expression is regulated by IL-4 family cytokines (IL-4, IL-13, IL-3, GM-CSF) in human monocytes (Musso et al., 1994; Hiremath et al., 2004), and by stem cell factor (SCF) and PMA in the human megakaryoblastic cell line MO7e (Grgurevich et al., 1997). The consistent increases in CHK mRNA and protein levels induced by the cytokines demonstrate that, in both blood cell types, transcriptional control of CHK stimulated by cytokines may play important roles in regulating CHK levels (Grgurevich et al., 1997). However, CHK induction can be inhibited or reversed by treatment of IFN-γ, which controls protein synthesis (Hiremath et al., 2004).

In colon cancer cells, the downregulation of CHK expression was associated with a high level of promoter methylation of CHK, which has been shown to be associated with enhanced levels of DNA methyltransferases (DNMTs). The hypermethylation of CHK promoters by DNMT promotes the oncogenic properties of colon cancer cells (Chüeh et al., 2021; Zhu et al., 2021).

## 9 Closing remarks

Unlike CSK, the roles that CHK plays are still largely unknown. Exploring potential CHK substrate(s) and verifying its roles in the signaling pathways are important in defining the exact physiological roles CHK plays. Furthermore, it is important to define roles of different isoforms of CHK. Depending on the cellular context, CHK may act as a tumor suppressor, possibly *via* inhibiting SFK signaling pathways, or act as a protooncogene, activating MAPK signaling pathways. Besides, CHK absence or its promoter methylation in colon cancer and brain cancer suggests its potential as a molecular biomarker in these cancer types and holds promise to become a novel cancer diagnosis standard alone or in combination with other molecules based on further clinical investigations. The ability of CHK to suppress tumorigenicity in a variety of cancers also holds promise for using CHK as an effective therapeutic intervention.

## Abbreviations

SH1 (2, 3), src homology 1 (2, 3); SFK, Src family kinase; CSK, C-terminal Src kinase; CHK, CSK-homologous kinase; KESTREL, kinase substrate tracking and elucidation; SHPS-1, Src homology 2 domain containing protein tyrosine phosphatase substrate-1; SCF/KL, stem cell factor/kit ligand; PI3K, phosphatidylinositol 3-kinase; PLCγ-1, phospholipase Cγ-1; DNMT, DNA methyltransferase; IL-3 (4), interleukin-3 (4); VSMC, vascular smooth muscle cell; MAPK, mitogen-activated protein kinase; Cbp, CSK binding protein; PTP1B, protein tyrosine phosphatase 1B; SHP-2, SH2 domain-containing protein-tyrosine phosphatase-2; Grb2, growth factor receptor-bound protein 2; ras-GAP, Ras GTPase activating protein; VLA5, integrin very late antigen.

## References

[B1] Advani G. Lim Y. C. Catimel B. Lio D. Ng N. Chüeh A. C. (2017). Csk-homologous kinase (Chk) is an efficient inhibitor of Src-family kinases but a poor catalyst of phosphorylation of their C-terminal regulatory tyrosine. Cell Commun. Signal. 15 (1), 29. 10.1186/s12964-017-0186-x 28784162 PMC5547543

[B2] Avraham S. Jiang S. Ota S. Fu Y. Deng B. Dowler L. L. (1995). Structural and functional studies of the intracellular tyrosine kinase MATK gene and its translated product. J. Biol. Chem. 270 (4), 1833–1842. 10.1074/jbc.270.4.1833 7530249

[B3] Ayrapetov M. K. Nam N. H. Ye G. Kumar A. Parang K. Sun G. (2005). Functional diversity of Csk, Chk, and Src SH2 domains due to a single residue variation. J. Biol. Chem. 280 (27), 25780–25787. 10.1074/jbc.M504022200 15890649

[B4] Bougeret C. Jiang S. Keydar I. Avraham H. (2001). Functional analysis of Csk and CHK kinases in breast cancer cells. J. Biol. Chem. 276 (36), 33711–33720. 10.1074/jbc.M104209200 11445575

[B5] Brinkley P. M. Class K. Bolen J. B. Penhallow R. C. (1995). Structure and developmental regulation of the murine ctk gene. Gene 163 (2), 179–184. 10.1016/0378-1119(95)00352-7 7590263

[B6] Chong Y. P. Chan A. S. Chan K. C. Williamson N. A. Lerner E. C. Smithgall T. E. (2006). C-terminal Src kinase-homologous kinase (CHK), a unique inhibitor inactivating multiple active conformations of Src family tyrosine kinases. J. Biol. Chem. 281 (44), 32988–32999. 10.1074/jbc.M602951200 16959780

[B7] Chong Y. P. Mulhern T. D. Zhu H. J. Fujita D. J. Bjorge J. D. Tantiongco J. P. (2004). A novel non-catalytic mechanism employed by the C-terminal Src-homologous kinase to inhibit Src-family kinase activity. J. Biol. Chem. 279 (20), 20752–20766. 10.1074/jbc.M309865200 14985335

[B8] Chow L. M. Davidson D. Fournel M. Gosselin P. Lemieux S. Lyu M. S. (1994a). Two distinct protein isoforms are encoded by ntk, a csk-related tyrosine protein kinase gene. Oncogene 9 (12), 3437–3448. 7970703

[B9] Chow L. M. Jarvis C. Hu Q. Nye S. H. Gervais F. G. Veillette A. (1994b). Ntk: A csk-related protein-tyrosine kinase expressed in brain and T lymphocytes. Proc. Natl. Acad. Sci. U. S. A. 91 (11), 4975–4979. 10.1073/pnas.91.11.4975 8197166 PMC43912

[B10] Chüeh A. C. Advani G. Foroutan M. Smith J. Ng N. Nandurkar H. (2021). CSK-homologous kinase (CHK/MATK) is a potential colorectal cancer tumour suppressor gene epigenetically silenced by promoter methylation. Oncogene 40 (17), 3015–3029. 10.1038/s41388-021-01755-z 33767439

[B11] Davidson D. Chow L. M. Veillette A. (1997). Chk, a Csk family tyrosine protein kinase, exhibits Csk-like activity in fibroblasts, but not in an antigen-specific T-cell line. J. Biol. Chem. 272 (2), 1355–1362. 10.1074/jbc.272.2.1355 8995444

[B12] Dokmanovic M. Wu Y. Shen Y. Chen J. Hirsch D. S. Wu W. J. (2014). Trastuzumab-induced recruitment of Csk-homologous kinase (CHK) to ErbB2 receptor is associated with ErbB2-Y1248 phosphorylation and ErbB2 degradation to mediate cell growth inhibition. Cancer Biol. Ther. 15 (8), 1029–1041. 10.4161/cbt.29171 24835103 PMC4119070

[B13] Fu Y. Zagozdzon R. Avraham R. Avraham H. K. (2006). CHK negatively regulates Lyn kinase and suppresses pancreatic cancer cell invasion. Int. J. Oncol. 29 (6), 1453–1458. 10.3892/ijo.29.6.1453 17088984

[B14] Grgurevich S. Linnekin D. Musso T. Zhang X. Modi W. Varesio L. (1997). The Csk-like proteins Lsk, Hyl, and Matk represent the same Csk homologous kinase (Chk) and are regulated by stem cell factor in the megakaryoblastic cell line MO7e. Growth factors (Chur, Switz. 14 (2-3), 103–115. 10.3109/08977199709021514 9255603

[B15] Grgurevich S. Mikhael A. McVicar D. W. (1999). The Csk homologous kinase, Chk, binds tyrosine phosphorylated paxillin in human blastic T cells. Biochem. Biophys. Res. Commun. 256 (3), 668–675. 10.1006/bbrc.1999.0398 10080957

[B16] Hamaguchi I. Iwama A. Yamaguchi N. Sakano S. Matsuda Y. Suda T. (1994). Characterization of mouse non-receptor tyrosine kinase gene, HYL. Oncogene 9 (11), 3371–3374. 7936664

[B17] Hamaguchi I. Yamaguchi N. Suda J. Iwama A. Hirao A. Hashiyama M. (1996). Analysis of CSK homologous kinase (CHK/HYL) in hematopoiesis by utilizing gene knockout mice. Biochem. Biophys. Res. Commun. 224 (1), 172–179. 10.1006/bbrc.1996.1003 8694808

[B18] Hirao A. Hamaguchi I. Suda T. Yamaguchi N. (1997). Translocation of the Csk homologous kinase (Chk/Hyl) controls activity of CD36-anchored Lyn tyrosine kinase in thrombin-stimulated platelets. EMBO J. 16 (9), 2342–2351. 10.1093/emboj/16.9.2342 9171348 PMC1169835

[B19] Hirao A. Huang X. L. Suda T. Yamaguchi N. (1998). Overexpression of C-terminal Src kinase homologous kinase suppresses activation of Lyn tyrosine kinase required for VLA5-mediated Dami cell spreading. J. Biol. Chem. 273 (16), 10004–10010. 10.1074/jbc.273.16.10004 9545346

[B20] Hiremath M. M. Mikhael A. I. Taylor L. S. Musso T. McVicar D. W. (2004). Complex regulation of the Csk homologous kinase (Chk) by IL-4 family cytokines and IFN-gamma in human peripheral blood monocytes. Mol. Immunol. 41 (9), 901–910. 10.1016/j.molimm.2004.04.025 15261462

[B21] Ia K. K. Jeschke G. R. Deng Y. Kamaruddin M. A. Williamson N. A. Scanlon D. B. (2011). Defining the substrate specificity determinants recognized by the active site of C-terminal Src kinase-homologous kinase (CHK) and identification of β-synuclein as a potential CHK physiological substrate. Biochemistry 50 (31), 6667–6677. 10.1021/bi2001938 21699177 PMC3156789

[B22] Imamoto A. Soriano P. (1993). Disruption of the csk gene, encoding a negative regulator of Src family tyrosine kinases, leads to neural tube defects and embryonic lethality in mice. Cell 73 (6), 1117–1124. 10.1016/0092-8674(93)90641-3 7685657

[B23] Jhun B. H. Rivnay B. Price D. Avraham H. (1995). The MATK tyrosine kinase interacts in a specific and SH2-dependent manner with c-Kit. J. Biol. Chem. 270 (16), 9661–9666. 10.1074/jbc.270.16.9661 7536744

[B24] Kaneko Y. Nonoguchi K. Fukuyama H. Takano S. Higashitsuji H. Nishiyama H. (1995). Presence of alternative 5' untranslated sequences and identification of cells expressing ctk transcripts in the brain and testis. Oncogene 10 (5), 945–952. 7898936

[B25] Kawabuchi M. Satomi Y. Takao T. Shimonishi Y. Nada S. Nagai K. (2000). Transmembrane phosphoprotein Cbp regulates the activities of Src-family tyrosine kinases. Nature 404 (6781), 999–1003. 10.1038/35010121 10801129

[B26] Kim S. O. Avraham S. Jiang S. Zagozdzon R. Fu Y. Avraham H. K. (2004). Differential expression of Csk homologous kinase (CHK) in normal brain and brain tumors. Cancer 101, 1018–1027. 10.1002/cncr.20442 15329911

[B27] Kim S. Zagozdzon R. Meisler A. Baleja J. D. Fu Y. Avraham S. (2002). Csk homologous kinase (CHK) and ErbB-2 interactions are directly coupled with CHK negative growth regulatory function in breast cancer. J. Biol. Chem. 277 (39), 36465–36470. 10.1074/jbc.M206018200 12122014

[B28] Klages S. Adam D. Class K. Fargnoli J. Bolen J. B. Penhallow R. C. (1994). Ctk: A protein-tyrosine kinase related to csk that defines an enzyme family. Proc. Natl. Acad. Sci. U. S. A. 91 (7), 2597–2601. 10.1073/pnas.91.7.2597 7511815 PMC43416

[B29] Lee B. C. Avraham S. Imamoto A. Avraham H. K. (2006). Identification of the nonreceptor tyrosine kinase MATK/CHK as an essential regulator of immune cells using Matk/CHK-deficient mice. Blood 108 (3), 904–907. 10.1182/blood-2005-12-4885 16574955 PMC1895851

[B50] Lennartsson J. Rönnstrand L. (2012). Stem cell factor receptor/c-Kit: from basic science to clinical implications. Physiol. Rev. 92 (4), 1619–1649. 10.1152/physrev.00046.2011 23073628

[B30] McVicar D. W. Lal B. K. Lloyd A. Kawamura M. Chen Y. Q. Zhang X. (1994). Molecular cloning of lsk, a carboxyl-terminal src kinase (csk) related gene, expressed in leukocytes. Oncogene 9 (7), 2037–2044. 7516063

[B31] Mikkola E. T. Bergman M. (2003). Conserved hydrophobicity in the SH2-kinase linker is required for catalytic activity of Csk and CHK. FEBS Lett. 544 (1-3), 11–14. 10.1016/s0014-5793(03)00405-8 12782282

[B32] Musso T. Varesio L. Zhang X. Rowe T. K. Ferrara P. Ortaldo J. R. (1994). IL-4 and IL-13 induce Lsk, a Csk-like tyrosine kinase, in human monocytes. J. Exp. Med. 180 (6), 2383–2388. 10.1084/jem.180.6.2383 7964512 PMC2191790

[B33] Nada S. Yagi T. Takeda H. Tokunaga T. Nakagawa H. Ikawa Y. (1993). Constitutive activation of Src family kinases in mouse embryos that lack Csk. Cell 73 (6), 1125–1135. 10.1016/0092-8674(93)90642-4 8513497

[B34] Nakayama Y. Kawana A. Igarashi A. Yamaguchi N. (2006). Involvement of the N-terminal unique domain of Chk tyrosine kinase in Chk-induced tyrosine phosphorylation in the nucleus. Exp. Cell Res. 312 (12), 2252–2263. 10.1016/j.yexcr.2006.03.021 16707123

[B35] Nakayama Y. Yamaguchi N. (2005). Multi-lobulation of the nucleus in prolonged S phase by nuclear expression of Chk tyrosine kinase. Exp. Cell Res. 304 (2), 570–581. 10.1016/j.yexcr.2004.11.027 15748901

[B36] Oda H. Kumar S. Howley P. M. (1999). Regulation of the Src family tyrosine kinase Blk through E6AP-mediated ubiquitination. Proc. Natl. Acad. Sci. U. S. A. 96 (17), 9557–9562. 10.1073/pnas.96.17.9557 10449731 PMC22247

[B37] Okada M. Nada S. Yamanashi Y. Yamamoto T. Nakagawa H. (1991). CSK: A protein-tyrosine kinase involved in regulation of src family kinases. J. Biol. Chem. 266 (36), 24249–24252. 10.1016/s0021-9258(18)54220-4 1722201

[B38] Pan Q. Qiao F. Gao C. Norman B. Optican L. Zelenka P. S. (2011). Cdk5 targets active Src for ubiquitin-dependent degradation by phosphorylating Src(S75). Cell. Mol. Life Sci. 68 (20), 3425–3436. 10.1007/s00018-011-0638-1 21442427 PMC3167940

[B39] Price D. J. Rivnay B. Fu Y. Jiang S. Avraham S. Avraham H. (1997). Direct association of Csk homologous kinase (CHK) with the diphosphorylated site Tyr568/570 of the activated c-KIT in megakaryocytes. J. Biol. Chem. 272 (9), 5915–5920. 10.1074/jbc.272.9.5915 9038210

[B40] Radhakrishnan Y. Shen X. Maile L. A. Xi G. Clemmons D. R. (2011). IGF-I stimulates cooperative interaction between the IGF-I receptor and CSK homologous kinase that regulates SHPS-1 phosphorylation in vascular smooth muscle cells. Mol. Endocrinol. 25 (9), 1636–1649. 10.1210/me.2011-0035 21799000 PMC3165910

[B41] Samokhvalov I. Hendrikx J. Visser J. Belyavsky A. Sotiropolous D. Gu H. (1997). Mice lacking a functional chk gene have no apparent defects in the hematopoietic system. Biochem. Mol. Biol. Int. 43 (1), 115–122. 10.1080/15216549700203881 9315289

[B42] Turner C. E. Glenney J. R. Jr Burridge K. (1990). Paxillin: A new vinculin-binding protein present in focal adhesions. J. Cell Biol. 111 (3), 1059–1068. 10.1083/jcb.111.3.1059 2118142 PMC2116264

[B43] Yamaguchi N. Nakayama Y. Urakami T. Suzuki S. Nakamura T. Suda T. (2001). Overexpression of the csk homologous kinase (chk tyrosine kinase) induces multinucleation: A possible role for chromosome-associated chk in chromosome dynamics. J. Cell Sci. 114 (9), 1631–1641. 10.1242/jcs.114.9.1631 11309195

[B44] Yamashita H. Avraham S. Jiang S. Dikic I. Avraham H. (1999). The Csk homologous kinase associates with TrkA receptors and is involved in neurite outgrowth of PC12 cells. J. Biol. Chem. 274 (21), 15059–15065. 10.1074/jbc.274.21.15059 10329710

[B45] Yeatman T. J. (2004). A renaissance for SRC. Nat. Rev. Cancer 4 (6), 470–480. 10.1038/nrc1366 15170449

[B46] Zagozdzon R. Kaminski R. Fu Y. Fu W. Bougeret C. Avraham H. K. (2006). Csk homologous kinase (CHK), unlike Csk, enhances MAPK activation via Ras-mediated signaling in a Src-independent manner. Cell. Signal. 18 (6), 871–881. 10.1016/j.cellsig.2005.07.016 16168623

[B47] Zhu S. Bjorge J. D. Cheng H. C. Fujita D. J. (2008). Decreased CHK protein levels are associated with Src activation in colon cancer cells. Oncogene 27 (14), 2027–2034. 10.1038/sj.onc.1210838 17934522

[B48] Zhu S. Zhu Y. Wang Q. Zhang Y. Guo X. (2021). CHK methylation is elevated in colon cancer cells and contributes to the oncogenic properties. Front. Cell Dev. Biol. 9, 708038. 10.3389/fcell.2021.708038 34268315 PMC8276677

[B49] Zrihan-Licht S. Lim J. Keydar I. Sliwkowski M. X. Groopman J. E. Avraham H. (1997). Association of csk-homologous kinase (CHK) (formerly MATK) with HER-2/ErbB-2 in breast cancer cells. J. Biol. Chem. 272 (3), 1856–1863. 10.1074/jbc.272.3.1856 8999872

